# Role of Angiopoietin-2 in Regulating Growth and Vascularity of Astrocytomas

**DOI:** 10.1155/2010/659231

**Published:** 2010-05-11

**Authors:** Gelareh Zadeh, Keyvan Koushan, Qian Baoping, Patrick Shannon, Abhijit Guha

**Affiliations:** ^1^Arthur & Sonia Labatts Brain Tumor Center, Hospital for Sick Children, University of Toronto, Toronto, Canada M5G 2G4; ^2^Department of Pathology, Western Hospital, University of Toronto, Toronto, Canada M5G 2G4; ^3^Division of Neurosurgery, Western Hospital, University of Toronto, Toronto, Canada M5G 2G4

## Abstract

Angiopoietins and Tie2 are angiogenic-specific ligand and receptor complex that have been shown to play a critical role in tumor angiogenesis. Angiopoietin-2 (Ang2) is one of four ligands for receptor Tie2 and it is the naturally occurring antagonist to Tie2, inhibiting the action of Angiopoietin-1 (Ang1). Over the last decade, significant research has focused on elucidating the role of Ang2 in cancer biology and its exact role in tumor angiogenesis remains elusive. In this study we have focused on establishing the role of Ang2 in angiogenesis of malignant astrocytomas. We have demonstrated that Ang2 significantly enhances the vascular growth of malignant astrocytomas and constant upregulation of Ang2 throughout all phases of tumor growth generates abnormal vascular structures that are not typically seen in human astrocytomas, suggesting that Ang2 plays a tumor stage-dependent role and is not a consistently elevated throughout all growth stages of malignant astroctyomas.

## 1. Introduction

Based on the originally proposed paradigm by Holash et al., Angiopoietin-2 (Ang2) works in concert with VEGF to promote neoangiogenesis, and in the absence of VEGF, vessels that have been destabilized by Ang2 will undergo apoptosis and regress. Ang2 is the naturally occurring antagonist to Ang1 and inhibits Ang1-induced activation of Tie2/TEK. Though there has been numerous biochemical data to support this paradigm [1–7], there is sufficient data to suggest a more complex role for Ang2. For example, at high concentrations Ang2 acts as an agonist of Tie2/TEK, providing a prosurvival signal to endothelial cell (EC), which is a similar function as Ang1 [1]. Although all tumor models show an upregulation of Ang2, its role in tumor angiogenesis has proven to be quite complex and variable, depending on the tumor model investigated [8–14]. Ang2 upregulation is seen primarily in EC of small cell lung cancer, hepatocellular carcinoma, neuroblastoma, gastric cancer, colon cancer, and Kaposi sarcoma, with Ang2 being associated with poor prognosis in many of these tumors [8–16]. 

Upregulation of Ang2 along with VEGF upregulation suggests that vessel destabilization by Ang2 is a critical step required to allow for VEGF-induced neoangiogenesis. In astrocytomas, Ang2 has been found to be upregulated in GBMs compared to LGAs and NB [11, 17–19]. The source of Ang2 is mainly reported to be the EC; however, one study and our own unpublished data suggest that Ang2 may also be expressed by malignantly transformed astrocytoma cells [11]. A noteworthy observation made by Stratmann et al. is that expression of Ang2 appears to be vessel size- or vessel type-dependent [20]. Ang2 expression was confined to EC of smaller vessels and not seen in larger vessels suggesting that Ang2 promotes *in-situ *angiogenesis and is more intimately involved with capillary-like vascular structures in tumors [18, 20]. 

A more recent study identifies Ang2 as a marker of tumor cell invasion in high-grade astrocytomas, with little Ang2 expression seen in the center of human GBM compared to the invasive peripheral edge of the tumors where Ang2 is expressed by both the vascular and neural elements [13]. They also found that upregulation of Ang2 in U87 xenografts had a pronounced invasive phenotype compared to the parental U87MG xenografts that had no Ang2 expression [13]. They propose that Ang2 confers a more invasive phenotype to the tumor cells via either activation of MMP-2, independent of Tie2/TEK receptor activation, or perhaps via activation of integrins [13]. 

In this study, we have focused on deciphering the distinct contribution of Ang2 to GBM angiogenesis and vessel development. We have found that Ang2 promotes vascular growth of GBMs. Additionally, Ang2 induces vascular architectural changes that are pathological and aberrant in comparison to control tumor vessels. This aberrancy in vasculature is not seen in human GBMs, which suggests that Ang2 is not constantly upregulated in human tumors and alludes to a stage-dependent upregulation of Ang2.

## 2. Materials and Methods

### 2.1. Cells and Reagents

Established human U87-MG GBM cells were obtained from American Type Culture Collection (ATCC, Rockville, MD) and U373-MG cell lines were a gift from B. Westermark (Uppsala, Sweden). These GBM lines were chosen as they provide variability in their degree of baseline Angiopoietin and VEGF-A expression [17], in addition to variable tumorigenicity potential and differences in genetic aberrations. They were maintained in Dulbecco's minimal essential medium (DMEM) (Cellgro, Herndon, VA) supplemented with 10% FBS and penicillin-streptomycin antibiotics.

### 2.2. Stable Clones

#### 2.2.1. Constitutively Overexpressing Clones

Full-length human ANG2 cDNA (a gift from K. Alitalo, Helsinki, Finland) was subcloned into the pSec vector (Invitrogen) to allow generation of Myc-Histidine epitope-tagged constructs. The Ang-Myc/HIS sequence was subcloned into the BamH1 and EcoR1 sites of the pCAGG vector that contained a CMV promoter along with a chicken *β*-actin enhancer element. Stable cell lines expressing Ang2, were generated by transfection of the vector “pCAGG-Ang-Myc/HIS-Zeocin” into U87 and U373 GBM lines using Lipofectamine 2000 (Gibco/BRL) as per the manufacturer's instructions. Twenty stable clones, selected with 1mg/ml of Zeocin (Invitrogen), were examined for Ang2 expression by western blot analysis as described below. Two single clones with highest expression of Ang2 above baseline parental levels, as well as one pooled clone of Ang2 were selected for each of the three GBM lines (U87:Ang2, U373:Ang2). Corresponding control stable cell lines were generated using empty-vector transfectants.

#### 2.2.2. Tetracycline Inducible Clones Overexpressing Angiopoietins

As described previously, stable Tet-Off U87-MG cells were established [21]. Briefly, U87-MG cell lines were transfected with pTet-Off (Clontech, Palo Alto) vector and stable clones selected and maintained in 1 mg/mL and 500 *μ*g/mL of G418, respectively. Thirty of the Tet-Off clones were assayed by transfecting with the reporter construct pTRE-LUC and subsequent examination of luciferase activity with a luciferase assay. The highest tetracycline inducible clone was selected to generate double stable Tet-Off cell lines (data not shown). Double stable Tet-Off U87-MG cell lines overexpressing Ang2 were generated by cotransfecting U87-MG:Tet-Off stable cells with pTRE-Ang2 with the pTK-Puromycin vector. Stable clones were selected in 3 mg/mL of Puromycin, and twenty clones were tested for induction of Ang2 expression by immunoprecipitation followed by western blotting, as described below. For control U87-MGTet-off double stable cell lines, pTRE-Red vector expressing the ds-RED fluorescent protein was used. *In vitro* testing of tetracycline induction of Ang2 expression was determined using varying doses of Doxycycline, with the most tightly regulated clones expressing Ang2 selected for *in vivo* experiments.

### 2.3. In Vivo Tumor Models

#### 2.3.1. Subcutaneous Models

Subcutaneous xenografts were generated by growing U87-MG stable clones overexpressing Ang2 in the flanks of NOD-SCID mice. For each stable clone, seven mice were injected with 10^7^ cells suspended in 300 *μ*L of PBS, with five mice injected with control empty vector transfectants. Tumor growth was measured biweekly, using calipers by two observers in a blinded fashion. Tumor volume was calculated using the formula: (diameter^2^× length)/2. As per animal protocol, mice were sacrificed by cervical dislocation after 100 mg/kg BrDU injection (Sigma-Aldrich). Tumors were cut in cross sections, with two cross-sections kept in formaldehyde for paraffin blocks and immunohistochemical analysis and the remaining tumor stored in liquid nitrogen. All in vivo tumor models were repeated in duplicate.

#### 2.3.2. Intracranial Models

For orthotopic xenograft models, Tet-Off regulated human U87-MG:Ang2 cells (10^6^) were stereotactically injected 3 mm deep into the frontal cortex of NOD-SCID mice. Mice were treated with Dox in the drinking water with three doses of 0, 1, and 10 mg/mL. These doses were selected based on prior published studies demonstrating that Dox crosses the blood-brain barrier efficiently to regulate gene expression in the brain [21]. When animals exhibited symptoms consistent with failure to thrive or raised intracranial pressure, the mice were sacrificed by perfusion fixation after BrDU injection and tail vein injection of 2% Evans Blue solution (2 mL/kg) to determine intraluminal blood flow and vessel permeability. The time interval between the injection of Evans Blue and the perfusion and killing of the mice was approximately 30 minutes. All in vivo experiments were repeated in duplicate.

### 2.4. Tumor Vascularity

Four different tumor portions were each cut at 5 *μ*m consecutive paraffin sections and stained with the EC specific marker anti-FactorVIII (DAKO; 1  :  2000), followed by detection with an avidin-biotin complex method-3,3′-diaminobenzidine (VectaStain Elite; Vector Laboratories) system. Microvessel density (MVD) was calculated by counting the number of hollow lumen vessels in ten high-power fields (HPF:500x) and in five HPF at vascular “hot spots”. Areas that included abnormal vascular structures, such as glomeruloid bodies, were not included in the MVD count as the functional status of these vascular units in both human and xenograft tumors is not known. All analyses were carried out using the MicroComputer Image Device (MCID-Imaging Research, Inc.) linked to a color CCD camera (Sony DXC 970 MD) mounted on a transmitted-light microscope (Zeiss Axioskop). IHC for EC and SMC staining was performed using FactorVIII antibody and smooth muscle antigen (SMA) staining.

### 2.5. Immunohistochemistry

Standard hematoxylin and eosin (H&E) staining and immunohistochemical analysis was performed on 5 *μ*m tissue sections from paraffin embedded tissue blocks. Primary antibodies used include: FactorVIII (rabbit polyclonal antibody #A0082; DAKO; used at 1  :  2500) and a polyclonal goat anti-Ang2 antibody (1  :  200 and 1  :  400, Santa Cruz). Secondary antibody was a goat antimouse antibody (Zymed) used at 1  :  200, and antigens were detected using the avidin-biotin complex method (Vector Laboratories) and diaminobenzidine substrate. 

### 2.6. Statistical Analysis

All analyses were completed using StatView 4.1 for the Macintosh (Abacus Concepts, Berkeley, CA). All errors were calculated as the standard error of the mean (S.E.M.). One-tailed Student's *t*-test was used to compare means (two sample, unequal variance) with *P* < .05 considered statistically significant.

## 3. Results

### 3.1. Overexpression of Ang2 in GBM Cell Lines

**Figure 1:**
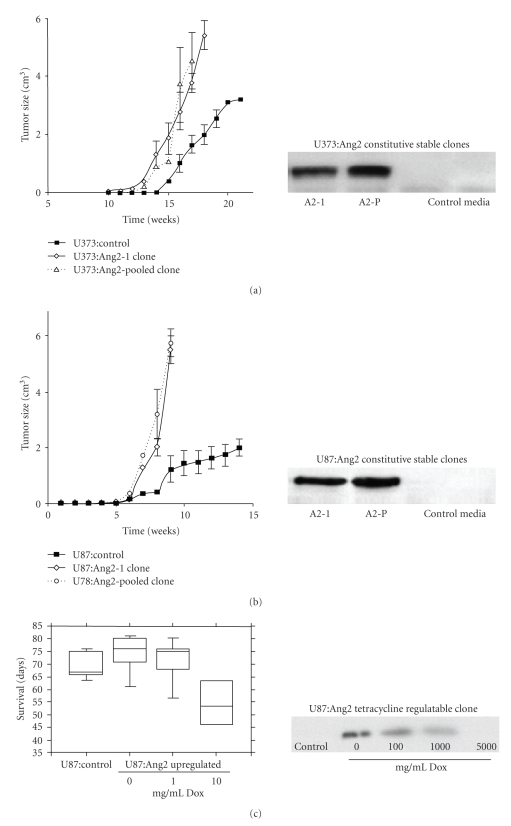
Effect of Ang2 over-expression on growth of GBM xenografts. Stable clones of U87 and U373 were generated to over-express Ang2 constitutively. Neither of the cell lines expresses Ang2 at baseline. One highest expressing clone and one pooled clone of each cell line was grown as subcutaneous models. Ang2 restricted tumor growth in U373 (a) tumors while it conferred a growth advantage in U87 tumors (b). Similarly, a growth advantage was maintained in U87 intracranial xenografts as evidenced by a significantly lowered survival time of mice with these grafts compared to mice with control tumors, and this increased tumor growth was dose dependent on Ang2 (c).

Parental U87MG and U373MG cells have no detectable Ang2 (Figures 1(a) and 1(b)) [17]. Overexpression of Ang2 did not alter the *in vitro* proliferation rate, morphology, or the VEGF expression of the cells compared to parental controls (data not shown). Stable transfectants overexpressing the highest levels of Ang2 (A2-1) and one pooled (A2-p) clone were selected for subsequent experiments (Figures 1(a) and 1(b)). Tet-Off regulated Ang2 stable clones were also established in U87MG cells, with the most tightly regulated clones selected for *in vivo* studies (Figure 1(c)). In the U87MG:Ang2 Tet-Off clone, Dox at 5000 ng/mL was sufficient to decrease Ang2 expression to undetectable levels, as seen in control cell lines (Figure 1(c)).

### 3.2. Effect of Ang2 on Tumor Growth and Proliferation

We assessed the impact of Ang2 on the growth of GBM xenografts in both subcutaneous (s.c). and intracranial (i.c.) tumor models using stable cell lines of U87MG and U373MG overexpressing Ang2 (Figure 1(b)). In s.c. xenografts, Ang2 overexpression resulted in a significantly faster tumor growth and larger final tumor size compared to controls (Figure 1(b) and Table 1 **)**. In i.c. xenografts, Ang2 conferred a growth advantage as suggested by a significant decrease in survival and tumor proliferation (Figure 1(c) and Table 2). The response to Ang2 was dose-dependent with respect to survival, tumor proliferation, and vascularity (Figure 1(c) and Table 2). Mice treated with 0 mg/mL of Dox in the drinking water, hence those with xenografts expressing high levels of Ang2, had a significantly shorter survival time, and tumor proliferation was increased by 2.2-fold compared to the mice receiving either 1 or 10 mg/mL of Dox, which had comparable survival to controls (Figure 1(c) and Table 2 **)**. Ang2 is not expressed endogenously by U87MG cells (Figure 1(a)), therefore, addition of Dox can completely turn-off exogenous Ang2 and result in similar tumor growth and survival of mice as that of controls. 

**Figure 2:**
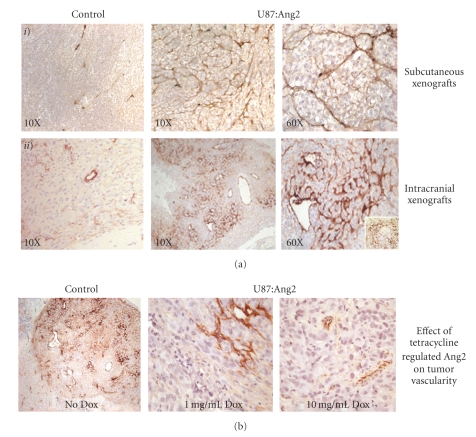
Effect of Ang2 on tumor vascularity. Immunohistochemical analysis of tumor vessels as determined by Factor VIII stains was performed on Ang2 upregulated xenografts. (a) *i*) subcutaneous and *ii*) intracranial tumors of U87MG:Ang2 demonstrated in addition to an increase in MVD, abnormal EC distribution with fine “cord” like-structures dispersed throughout the tumor, areas of EC whirling (inset *ii*) and dilated vessels. This process was seen in both the intracranial and subcutaneous xenografts and was dependent on the level of Ang2 expression. (b) Tumors with high levels of Ang2 expression (No Dox in the drinking water) had a small, highly infiltrative EC pattern together with dilated vessels, whereas these structural changes are lost with turning off of Ang2 using 1 mg/mL, and to a greater extent at 10 mg/mL, of Dox in the drinking water.

Ang2 upregulation resulted in increased MVD and altered vessel size and EC distribution in both s.c. and i.c. xenografts (Figure 2, Tables 1 and 2). Additionally, in both s.c. and i.c. U87MG:Ang2 xenografts, there was an abnormal vascular architecture, characterized by preponderance of small vessels, “cord”-like distribution of EC and whirling of EC present throughout the tumors, in addition to increased numbers of dilated vessels (Figure 2(a) *i* and *ii*). The alterations in the microvasculature were dependent on levels of Ang2 expression that were regulated by Dox in the i.c. U87MG:Ang2 models (Figure 1(b) and Table 1 **)**. At high levels of Ang2 (0 mg/mL Dox), a large number of dilated vessels were present, along with abnormal EC distribution throughout the tumor (Figure 2(b)). With Dox suppression (10 mg/mL Dox) of Ang2, EC distribution, vessel size, and the overall microvasculature architecture returned to similar structural patterns as is seen in control U87MG tumors (Figure 2(b) and Table 2). These vascular alterations have not been reported previously; though the recent publication by Hu et al. [13] and Lee et al. [19] makes the observation of impaired angiogenesis by Ang2, they do not report similar structural changes as ours on tumor vascularity.

## 4. Discussion

Astrocytomas angiogenesis is postulated to be highly tumor stage-dependant. At their initial growth phase, they coopt and parasitize existing host vessels in an attempt to support their growth, thus the first phase being independent of the tumor angiogenic process [18, 22–24]. The second growth phase begins when the host mounts a defensive response and the parasitized vascular supply regresses resulting in tumor hypoxia and necrosis, which in turn triggers upregulation of Ang2 and VEGF [23, 24]. Therefore, Ang2 appears to play a highly phase-dependent role in the progression of malignant astrocytomas. It plays a pivotal role in the cooption of host vessels in the initial phase; and in supporting *in-situ* tumor angiogenesis, while in the second phase it allows destabilization of mature vessels, by antagonizing Ang1-mediated Tie2/TEK activation, in order to facilitate mitogenic stimulation of ECs by VEGF and promoting tumor neovascularization [22, 24]. However, the role of Ang2 in tumor angiogenesis remains controversial. 

We have found that Ang2 upregulation, in both s.c. and i.c. xenografts, led to an increase in growth rate, final volume and proliferation of GBMs along with an increase in tumor angiogenesis. Ang2 upregulation resulted in an alteration of vascular structures, marked by abnormal EC distribution, with EC forming “cord” or capillary-like structures and areas of EC whorling present throughout the tumor, in addition to a high number of dilated vessels. In the model used in this study, there is constant upregulation of Ang2 throughout all stages and phase of GBM tumor growth, potentially providing a continual trigger for host vessel cooption and promoting *in-situ* angiogenesis, thereby increased tumor growth. 

On the other hand, Lee et al. have demonstrated a complex temporal and stage-dependent role for Ang2 [19]. They observe a bimodal expression pattern of Ang2 in astrocytomas and support the postulate that Ang2 is a vessel destabilize, seen at sites of tumor cell growth, tumor periphery, and around sites of necrosis, presumably to promote neoangiogenesis and support tumor cell growth [19]. However, quite contrary to what one would predict based on this observation, Lee et al. also found that Ang2 treatment of U87MG xenografts did not promote but rather restricted astrocytoma growth. Moreover, at first glance these results appear to be in opposition with our observations; however, on closer analysis, both findings can be seen as corroborative and together explain the complex tumor phase-dependent role of Ang2. Lee et al. treated the U87MG i.c. xenografts on day 4 after tumor implantation followed by biweekly injections of Ang2, whereas in our model Ang2 is upregulated constantly throughout all stages of tumor growth. The difference between the level and stages of Ang2 upregulation in the two models supports the postulate that Ang2 plays a highly tumor stage-dependent role. Another evidentiary data that Ang2 plays a stage dependent role in glioma angiogenesis is the fact that tumor vascular structures observed in our xenograft models are not evident in human GBM specimens, indicating that Ang2 is not upregulated throughout all stages of human GBMs, and most likely plays a very precise role at specific stages of GBM growth. 

The mechanism by which Ang2 causes abnormal vascular structures is not established. The abundant “cord” or capillary-like vessels in the U87MG:Ang2 xenografts may be a result of Ang2-mediated modulation of EC motility, migration, and invasion. Hu et al. demonstrate regions of Ang2-expressing tumors actively invading the brain, high levels of MMP-2 expression, and increased angiogenesis. The direct impact of Ang2 on EC invasion *in vivo *remains unknown. 

Additionally, Ang2 is known to influence the fate of new tumor vessels, differentiating them into capillaries versus arteries or venous structures. We observe dilated vessels throughout the GBM xenografts. The most likely explanation is that Ang2 presents an inhibitory signal, preventing Ang1-mediated maturation by of the newly formed tumor vessels. Taken together our data indicates that increased Ang2 promotes angiogenic growth of GBMs. Constant upregulation of Ang2 throughout all phases of tumor growth results in the abnormal vascular structures seen in our xenografts that are not present in human GBMs, suggesting that Ang2 upregulation in GBMs is very much a tumor stage dependent process and not constant throughout all stages of GBM growth. Future studies are required to decipher the precise temporal role of Ang2 and whether the combinatorial impact of other angiogenic cytokines with Ang2 can be used for therapeutic targets in treatment of GBMs.

## Figures and Tables

**Table 1:** Effect of Ang2 on subcutaneous xenograft models of GBM.

	U87MG:Ctl	U87MG:Ang2	U373:Ctl	U373MG:Ang2
	*n* = 8	*n* = 15	*n* = 15	*n* = 15
Final Tumor Size (cm^3^)	2.01	5.7 *	3.18	5.96
	(SEM 0.3)	(SEM 0.4)	(SEM 0.3)	(SEM 0.5)
		*P* = 4 × 10^−4^		*P* = 6 × 10^−5^

Proliferation Index	0.23	0.75*	0.042	0.28
		*P* = .0042		*P* = .001

MVD (vessels/HPF) mean of 10 counts	2.12	9.5*	3	3.9*
	(SEM 0.1)	(SEM 0.1)	(SEM 0.1)	(SEM 0.2)
		*P* = .001		*P* = .001

SEM = Standard Error of Mean

*indicates statistical significance.

**Table 2:** Effect of Ang2 on intracranial U87MG xenografts.

	U87MG:Ctl	U87MG:Ang2		
		no. Dox.	1 mg/mL Dox	10 mg/mL Dox
	*n* = 10	*n* = 10	*n* = 10	*n* = 10
Overall survival (days)	63.7	54.7*	71.7*	74.4
	(SEM = 2.3)	(SEM = 3.3)	(SEM = 2.3)	(SEM = 3.5)
		*P* = .015	*P* = .021	*P* = .0149

Proliferation index	0.043	0.094*	0.038*	0.035*
	(SEM = 0.01)	(SEM = 0.01)	(SEM = 0.00)	(SEM = 0.00)
		*P* = .0039	*P* = .3989	*P* = .4262

MVD (vessels/HPF) mean of 10 counts	5.8	8.8*	4.6*	4.0*
	(SEM = 0.478)	(SEM = 0.859)	(SEM = 0.616)	(SEM = 0.785)
		*P* = .0132	*P* = .038	*P* = .0189
